# Oxidized Regenerated Cellulose Mimicking a Retained Laparotomy Sponge

**DOI:** 10.1155/2022/4718457

**Published:** 2022-02-01

**Authors:** Lauren E. Karnolt, Andrea L. Buras, Thomas J. Rutherford, Matthew L. Anderson

**Affiliations:** ^1^Department of Obstetrics & Gynecology, University of South Florida Morsani College of Medicine, Tampa, FL 33623, USA; ^2^Tampa General Cancer Institute, Tampa, FL 33606, USA

## Abstract

Oxidized regenerated cellulose (ORC) is an absorbable hemostat commonly used during gynecologic surgery. We present a case in which ORC was used in a patient undergoing posterior pelvic exenteration with ureteroneocystostomy for excision of a malignant pelvic mass. At the conclusion of these procedures, the laparotomy pad count was reported as incomplete likely due to the large number of laparotomy pads used and changes in nursing staff. Abdominal radiographs were obtained to verify no pads were retained in the abdominal cavity. These identified a poorly defined radiolucency deep in the patient's pelvis, requiring the surgical incision be reopened. Upon reexploration, no evidence of a retained surgical sponge could be identified. However, ORC was identified at the site of the radiolucency in question. Radiographs of this material, once removed, confirmed its radiolucent appearance. This experience clearly demonstrates that oxidized regenerated cellulose can mimic a retained surgical sponge on intraoperative radiographs. Dissemination of this knowledge will hopefully help to avoid radiographic misidentification of ORC in the perioperative window and minimizing the risk of unnecessary surgical interventions in the future.

## 1. Introduction

Timely and accurate identification of retained surgical tools left erroneously within a patient is a key quality metric for nearly all surgical procedures. For many types of procedures, surgical teams accomplish this task by reconciling counts of instruments and other materials used over the course of a surgery. In situations where instrument counts cannot be reconciled, intraoperative radiographs are typically ordered as a means to determine whether a retained surgical item can be identified [1]. In situations where a count discrepancy still cannot be resolved, surgical reexploration is considered standard of care [2].

Although the incidence of retained surgical items following laparotomy is low, count discrepancies are not. One recent prospective study prospectively found at least 1 count discrepancy in 12.8% of 2,478 distinct counting episodes performed for 148 complex cases [3]. Although many discrepancies are easily and accurately resolved, reconciling mismatched counts can nonetheless require significant time and expense. In one study that reviewed more than 13,000 cases, the duration of time required to reconcile an incorrect count ranged from 1 to 90 minutes [1]. Furthermore, it is clear that the use of medical counts as a strategy to rule out retained surgical instruments is prone to error. In another study, 88% of retained surgical instruments occurred in cases in which the final surgical count was initially believed to be correct [2].

Topical hemostats are commonly used as an adjunct in diverse types of surgical procedures. In most circumstances, the use of these agents is recommended when suture ligation or electrocautery is not feasible, or to control minor bleeding [4–6]. Commercially available hemostats include preparations of microfibrillar collagen, gelatin hemostats, cyanoacrylate adhesives, or oxidized regenerated cellulose [7]. Of these, oxidized regenerated cellulose (ORC) has become an increasingly popular option, largely due to its ease of use and availability in structurally distinct preparations. Once placed in a surgical wound, ORC functions as a scaffold which promotes activation of the clotting cascade and platelet agglutination. Under normal circumstances, this material is absorbed within 7-14 days. Complications directly attributable to the use of ORC are considered rare [5, 6]. However, clinicians have previously documented a small number of cases where its use has resulted in findings suggestive of an abscess when patients have undergone postoperative imaging. Despite this, the radiographic appearance of most topical agents, including oxidized regenerated cellulose, remains largely undocumented.

Here, we present a case where the use of a structured, nonwoven formulation of ORC (known commercially as “Surgicel SNoW”) resulted in radiographic changes suggestive of a retained laparotomy sponge. This necessitated reexploration of a patient's abdomen and pelvis following a lengthy procedure.

## 2. Case Presentation

A 79-year-old woman presented for evaluation of postmenopausal vaginal bleeding. She had previously undergone hysterectomy for endometriosis at the age of 39. She had no prior history of cervical or vaginal dysplasia. Two months prior, she had experienced a cerebrovascular accident resulting in a mild expressive aphasia, right upper extremity weakness, and difficulty with ambulation. Due to the thrombotic nature of this event, she was started on therapeutic doses of an oral anticoagulant. Shortly thereafter, she began to experience persistent problems with vaginal bleeding. Pelvic ultrasound was performed, and patient was referred for consultation with a gynecologic oncologist. On pelvic exam, a large, maroon-colored mass was noted at the vaginal apex, approximately 7 cm in largest diameter. On bimanual exam, this mass was noted to be free from the pelvic sidewalls and rectum, although exam was limited by the patient's habitus. Multiple biopsies were obtained. These revealed only normal-appearing mesenchymal tissue. Serum tumor markers were evaluated and found to be abnormal: CA-125: 2633.3 U/mL (reference range, 0-35 U/ml); CA 19-9: 425 U/mL (reference range, 0-27 U/ml); CEA: 62.2 U/mL (reference range, 0-5 mg/ml).

Due to continued problems with vaginal bleeding and the need for long-term anticoagulation, the patient underwent laparotomy. No preoperative PET-CT scan was performed. At surgery, a highly fibrotic pelvic mass was identified at the vaginal apex. This mass was densely adherent to the rectosigmoid colon high within the pelvis, but with extension deep toward the pelvic floor along the left side of the patient's pelvis. Her abdomen and pelvis were otherwise grossly normal. Ultimately, posterior pelvic exenteration, bilateral ureterolysis, and end-descending colostomy were necessary to excise this mass, which was removed in one piece without rupture. Due to avulsion of the distal left ureter during debulking, the patient underwent ureteroneocystostomy. Multiple staging biopsies, along with total omentectomy, were also performed. Significant bleeding was encountered along the pelvic floor and left pelvic side wall at sites of surgical dissection. As a result, the patient received 9 units of packed red blood cells, 5 units of fresh frozen plasma, 4 packs of cryoprecipitate, and 3 packs of platelets intraoperatively. At the conclusion of these procedures, three pieces of structured, nonwoven ORC were placed along the denuded surfaces of pelvic floor and across the vaginal cuff.

**Figure 1:**
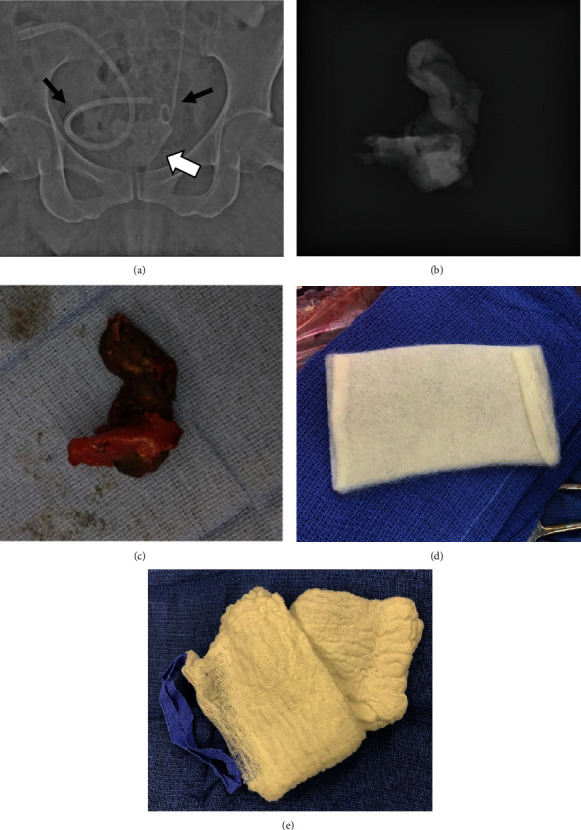
(a) Abdominal radiograph of patient prior to reexploration (white arrow indicates abnormal radiolucency which provoked exploration). Black arrows note the presence of a Jackson-Pratt drain (left) and ureteral stent (right) immediately adjacent to the radiolucent mass of oxidized regenerated cellulose. (b) Radiograph of nonwoven oxidized regenerated cellulose after its removal from the patient. (c) Color photograph of oxidized regenerated cellulose removed from patient's pelvis (1× magnification). (d) Color photograph of fresh oxidized regenerated cellulose (1× magnification). (e) Color photograph of an unused laparotomy pad (1/2× magnification).

As the patient's fascia was being reapproximated, the laparotomy pad count was reported as incomplete, likely due to the large number of pads used over the course of the surgery and changes in personnel with nursing shifts. Therefore, abdominal imaging was ordered.This revealed a radiolucent focus deep in the patient'spelvis (Figure 1(a)). Although the interpreting radiologist was made aware of the presence of ORC in the patient's pelvis, the radiologist felt unable to exclude a retained laparotomy sponge. Therefore, the patient's incision was opened sufficiently to allow her abdomen and pelvis to be thoroughly explored, adding approximately 75 minutes to the total procedure duration. No evidence of a retained laparotomy pad was identified. The hemostatic material, which had now absorbed small amounts of blood, was removed and radiographed (Figure 1(b)). Abdominopelvic radiographs were then repeated. These latter films demonstrated resolution of the radiolucency in question. Two fresh pieces of ORC were placed in the pelvis, and the patient's incision reapproximated and closed. No follow-up CT scan was performed.

Postoperatively, the patient remained hemodynamically stable. On the afternoon of the first postoperative day, she was noted to be poorly responsive with right-sided weakness. Imaging was obtained, which suggested new onset changes suggestive of cerebrovascular ischemia. She underwent percutaneous thrombectomy of the right basilar artery. The patient was discharged to home on postoperative day #16, without experiencing additional complications and improvement in residual aphasia and right upper extremity weakness. Final histopathologic evaluation of the tumor confirmed endometrioid carcinoma arising in endometriosis and extensive fibrosis. No evidence of metastatic disease was identified in specimens of omentum, left paracolic gutter, rectosigmoid, bowel mesentery, right pelvic lymph node, and appendix. After extensively counseling, she declined any form of adjuvant therapy. She remains alive and well, without evidence of recurrent disease 4 months following her surgery. Her most recent serum CA-125 level was found to be normal (9.3 U/mL).

## 3. Discussion

We present a case where following a mismatched surgical count, oxidized regenerated cellulose (ORC) placed in the patient's pelvis to optimize surgical hemostasis resulted in radiographic findings worrisome for a retained surgical sponge. Immediate reexploration of the patient's abdomen and pelvis uncovered no evidence of a retained sponge. Radiographs of the ORC removed directly from the patient's pelvis confirmed the radiolucent nature of this material (Figure 1(b)). Repeat films performed following the removal of this material confirmed that the radiolucent mass in question had been removed. Thus, there is little doubt that the abnormal radiolucent finding on her initial abdominal radiographs can be attributed to the use of this ORC.

Unfortunately, documentation of the radiographic appearance of most current surgical hemostatic agents remains sparse. As a result, the consulting radiologist in our case felt unable to exclude a retained surgical sponge. Prior manuscripts have described at least 2 cases where the presence of ORC had been misconstrued as a pelvic abscess when computed tomography was performed 1-2 weeks following a surgical procedure [8, 9]. Specifically, the presence of small gas or air bubbles within material iso-dense with adjacent tissue was described as highly suggestive of abscess [8–11]. These findings occurred at sites where ORC had been placed in a patient's pelvis. Similarly, the presence of ORC has shown to appear as an echogenic mass with “dirty” acoustic shadowing at the time of a postoperative ultrasound [9, 12].

Despite a thorough search of the English language medical literature using the search terms “oxidized regenerated cellulose,” “hemostat,” “radiograph,” “retained sponge,” “imaging,” and “X-ray” to interrogate PUBMED, we were unable to identify any other cases in which the appearance of ORC on plain radiographs has been previously described. Thus, documentation of our experience will hopefully create a precedent that can be referenced to avoid unnecessary surgical reexploration. We estimate that reexploration of our patient's abdomen added an additional 75 minutes of operative time to the patient's already complex procedure, likely compounding multiple other risks for experiencing adverse perioperative events.

Specific strengths of the current report include the fact the availability of radiographic images of the ORC immediately upon its removal from the patient as well as the use of intraoperative radiographs of the patient's pelvis after ORC had been removed. However, this report is also limited in that it summarizes our experiences with only one patient. It is reasonable to anticipate that the specific radiographic appearance of surgical hemostat will vary, depending on how a specific agent has been packed into a surgical site, as well as the volume of blood present. Thus, the radiographic appearance of this material could be reasonably expected to vary from case to case and may not appear exactly as shown in this report. This weakness underscores the fact that, ultimately, prevention of similar situations may require that more distinctive radiographic markers be incorporated into surgical hemostats so that these materials can be positively identified if patients undergo postoperative imaging.

## Data Availability

Data is available on request following approval by the institutional review board responsible for the University of South Florida College of Medicine.

## References

[1] Steelman V. M., Schaapveld A. G., Perkhounkova Y., Storm H. E., Mathias M. (2015). The hidden costs of reconciling surgical sponge counts. *AORN Journal*.

[2] Gawande A. A., Studdert D. M., Orav E. J., Brennan T. A., Zinner M. J. (2003). Risk factors for retained instruments and sponges after surgery. *The New England Journal of Medicine*.

[3] Greenberg C. C., Regenbogen S. E., Lipsitz S. R., Diaz-Flores R., Gawande A. A. (2008). The frequency and significance of discrepancies in the surgical count. *Annals of Surgery*.

[4] (2020). Topical hemostatic agents at time of obstetric and gynecologic surgery: ACOG committee opinion summary, number 812. *Obstetrics and Gynecology*.

[5] Morelli L., Morelli J., Palmeri M. (2015). Robotic surgery and hemostatic agents in partial nephrectomy: a high rate of success without vascular clamping. *Journal of Robotic Surgery*.

[6] Sharma J. B., Malhotra M., Pundir P. (2003). Laparoscopic oxidized cellulose (Surgicel) application for small uterine perforations. *International Journal of Gynaecology and Obstetrics*.

[7] Sundaram C. P., Keenan A. C. (2010). Evolution of hemostatic agents in surgical practice. *IJU: journal of the Urological Society of India*.

[8] Arnold A. C., Sodickson A. (2008). Postoperative Surgicel mimicking abscesses following cholecystectomy and liver biopsy. *Emergency Radiology*.

[9] Zhang F., Bonidie M. J., Ventrelli S. M., Furlan A. (2015). Intraovarian oxidized cellulose (Surgicel) mimicking acute ovarian pathology after recent pelvic surgery. *Radiol Case Reports*.

[10] Young S. T., Paulson E. K., McCann R. L., Baker M. E. (1993). Appearance of oxidized cellulose (Surgicel) on postoperative CT scans: similarity to postoperative abscess. *AJR. American Journal of Roentgenology*.

[11] Tam T., Harkins G., Dykes T., Gockley A., Davies M. (2014). Oxidized regenerated cellulose resembling vaginal cuff abscess. *JSLS: Journal of the Society of Laparoendoscopic Surgeons*.

[12] Heller H. T., Walker B. S., Sadow C. A., Frates M. C. (2017). Imaging appearance of topical haemostatic agents: pictorial review. *The British Journal of Radiology*.

